# nZVI-Based Nanomaterials Used for Phosphate Removal from Aquatic Systems

**DOI:** 10.3390/nano13030399

**Published:** 2023-01-18

**Authors:** Jonathan Suazo-Hernández, Pamela Sepúlveda, Lizethly Cáceres-Jensen, Jorge Castro-Rojas, Patricia Poblete-Grant, Nanthi Bolan, María de la Luz Mora

**Affiliations:** 1 Center of Plant, Soil Interaction and Natural Resources Biotechnology, Scientific and Biotechnological Bioresource Nucleus (BIOREN-UFRO), Universidad de La Frontera, Avenida Francisco Salazar 01145, Temuco 4780000, Chile; 2 Department of Chemical Sciences and Natural Resources, Universidad de La Frontera, Avenida Francisco Salazar 01145, Temuco 4811230, Chile; 3 University of Santiago of Chile (USACH), Physics Department, Faculty of Science and Faculty of Chemistry and Biology, Santiago 8320000, Chile; 4 Center for the Development of Nanoscience and Nanotechnology, CEDENNA, Santiago 9170022, Chile; 5 Physical & Analytical Chemistry Laboratory (PachemLab), Nucleus of Computational Thinking and Education for Sustainable Development (NuCES), Center for Research in Education (CIE-UMCE), Department of Chemistry, Metropolitan University of Educational Sciences, Santiago 776019, Chile; 6 Doctoral Program in Science of Natural Resources, Universidad de La Frontera, Avenida Francisco Salazar 01145, Temuco 4811230, Chile; 7 School of Agriculture and Environment, The University of Western Australia, Perth, WA 6009, Australia; 8 The UWA Institute of Agriculture, The University of Western Australia, Perth, WA 6009, Australia

**Keywords:** nanoscale zero-valent iron, nanocomposites, phosphorus, adsorption, aquatic environment

## Abstract

In the last decade, the application of nanoscale zero-valent iron (nZVI) has garnered great attention as an adsorbent due to its low cost, non-toxicity, high porosity, and BET-specific surface area. In particular, the immobilization of nZVI particles onto inorganic and organic substrates (nanocomposites) decreased its agglomeration, allowing them to be effective and achieve greater adsorption of pollutants than pristine nanoparticles (NPs). Although nZVI began to be used around 2004 to remove pollutants, there are no comprehensive review studies about phosphate removal from aquatic systems to date. For this reason, this study will show different types of nZVI, pristine nZVI, and its nanocomposites, that exist on the market, how factors such as pH solution, oxygen, temperature, doses of adsorbent, initial phosphate concentration, and interferents affect phosphate adsorption capacity, and mechanisms involved in phosphate removal. We determined that nanocomposites did not always have higher phosphate adsorption than pristine nZVI particles. Moreover, phosphate can be removed by nZVI-based nanoadsorbents through electrostatic attraction, ion exchange, chemisorption, reduction, complexation, hydrogen bonding, and precipitation mechanisms. Using the partition coefficient (PC) values, we found that sepiolite-nZVI is the most effective nanoadsorbent that exists to remove phosphate from aqueous systems. We suggest future studies need to quantify the PC values for nZVI-based nanoadsorbents as well as ought to investigate their phosphate removal efficiency under natural environmental conditions.

## 1. Introduction

Phosphorus (P) is an element used in industrial applications such as food processing, detergents, flame retardants, semi-conductors, and fertilizers [1]. Consequently, P can be released into natural aquatic systems by discharging wastewater treatment plants and industrial and agricultural runoff [2]. High P concentrations in aqueous systems can cause water eutrophication [3]. This process corresponds to the proliferation of algae and aquatic plants, causing deterioration of water (taste and smell) and changes in aquatic communities [4,5]. Algal blooms are a serious threat to the supply of drinking water and aquatic ecosystem sustainability [6]. Therefore, considering water quality is an essential resource for organisms and natural ecosystems, it has been established that the highest concentration of total P should be ≤50 µg L^−1^ in streams that flow into reservoirs and lakes or ≤25 µg L^−1^ within reservoirs and lakes [7].

Various techniques have been used to address this environmental problem and comply with established regulations, such as ion exchange, membrane separation, bioremediation, biological and chemical precipitation, and reverse osmosis [8,9]. However, these methods are generally expensive and have limitations such as low efficiency, the generation of toxic products, and difficulty in disposing of waste [10]. In this context, adsorption is considered a highly favorable technique compared to other technologies due to its ability to remove phosphate at low concentrations and produce a low amount of waste, being rapid, easy, environmentally friendly, and economical [11,12]. In recent years, the adsorption method has attracted great interest in connection with nanotechnology, which has made it possible to synthesize new and various adsorbents of a nanometric size (1–100 nm) [13]. Specifically, nanoscale zero-valent iron (nZVI) has become the most suitable material for water purification and the remediation of groundwater and soils contaminated with dyes, heavy metals, antibiotics, chlorinated organic compounds, halogenated organics, phenolic compounds, nitroaromatic compounds, and oxy-anions such as sulfate (SO_4_^2−^), arsenate (AsO_4_^3−^), selenate (SeO_4_^2−^), chromate (CrO_4_^2−^), dichromate (Cr_2_O_7_^2−^), nitrate (NO_3_^−^), and phosphate (PO_4_^3−^) [14,15]. nZVI is a material with high porosity and small particle size, contributing to its large specific surface area and high reactivity compared to bulk materials [16,17]. Furthermore, nZVI can be easily operated, non-toxic, and inexpensive, and due to its magnetic properties, spent nZVI can be easily recuperated from the solution using an external magnetic field, which eliminates the problem of recovery and secondary contamination [18].

**Figure 1:**
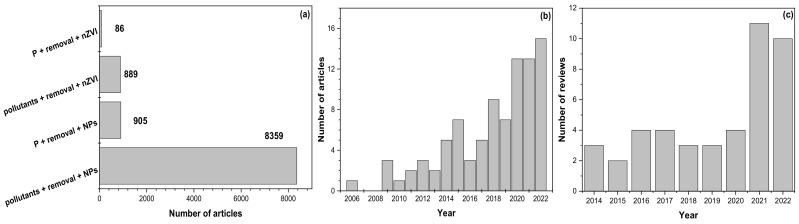
Search performed in the Scopus database (9 December 2022) using the words (**a**) phosphate (P), removal, nanoscale zero-valent iron (nZVI), pollutants, and nanoparticles (NPs). Increase in the number of articles by year based on words (**b**) P, removal, and nZVI. Increase in the number of reviews by year based on words (**c**) pollutants, removal, and nZVI.

nZVI in solution is quickly oxidized at a pH ≤ 7 and in the presence of oxygen, forming a core–shell structure, which consists of a metallic iron core covered by a thin layer of iron oxides/hydroxides [19,20]. This transformation enables phosphate anions, in addition to being adsorbed on the nZVI surface, to be removed through processes involving redox process, coagulation, precipitation, and co-precipitation by Fe^2+^/Fe^3+^ cations released from NPs [21,22]. In fact, of all the articles published about removing pollutants from aqueous systems using nZVI, about 10% have focused on phosphate, of which over 60% have been published in the past five years (Figure 1a,b), which shows the relevance, efficiency, and projection of nZVI particles to remove phosphate from aquatic systems. However, due to their magnetic properties, Van der Waals forces, and magnetic interaction, individual nZVI particles show easy agglomeration, sedimentation, and rapid oxidation, affecting their phosphate removal efficiency and preventing their application under natural conditions [2]. As an alternative to this problem, nZVI has been combined with inorganic and organic materials (clay minerals, noble metals, and carbon substrate) and stabilized with organic molecules (carboxymethyl cellulose, chitosan, polyvinylpyrrolidone, among others) [23]. In addition, they have been encapsulated in calcium–alginate beads [24] and few-layered graphene [25]. The latter has improved nZVI performance because it shows enhanced water stability, regeneration, and dispersion. As a result of these improvements, nZVI nanocomposites have usually demonstrated a higher maximum phosphate adsorption capacity (q_max_) than individual nZVI particles [26]. 

Although there are several reviews on pollutant removal from natural aqueous systems, groundwater, and wastewater using pristine nZVI and its nanocomposites (Figure 1c), no comprehensive review on phosphate removal using these nanoadsorbents has been published to date.

The goal of this review was to critically analyze information existing about pristine nZVI and its nanocomposites on phosphate adsorption under various experimental conditions. This research will help choose the most effective nZVI-based nanomaterials to remove phosphate from aqueous systems and potentially in the soil matrix.

## 2. Synthesis of Pristine nZVI

### 2.1. Top-Down Methods 

The top-down methods of nanomaterial synthesis correspond to the breakdown of iron minerals through mechanical processes such as milling, heating, engraving, and/or machining [27]. In particular, synthesized nZVI by top-down methods, which later on were used for phosphate removal, have varied only in synthesis temperature (°T) (Table 1) [28,29]. Liu et al. [28] synthesized nZVI by heating natural goethite [α-FeO(OH)] at 550 °C in the presence of H_2_ with a flow rate of 100 mL min^−1^ for 3 h. The nZVI obtained showed a particle size between 80 and 150 nm in length and between 5 and 30 nm in width, and the relatively large specific surface area was around 22 m^2^ g^−1^. Similarly, Zhang et al. [29] obtained nZVI by reducing natural limonite (FeO(OH)·nH_2_O) with a particle size < 74 μm under H_2_ conditions at different °T (i.e., 300–600 °C). The BET-specific surface area of nZVI at 300 °C was 37.52 m^2^ g^−1^ and reduced to 17.24 m^2^ g^−1^ when °T increased to 600 °C. The main limitations of the top-down method are the high operating cost, special equipment, and the low performance of nZVI.

### 2.2. Bottom-Up Methods

The bottom-up methods of nanomaterial synthesis are consistent in growing nanostructures atom by atom or molecule by molecule through positional assembly, chemical synthesis, and self-assembly [27]. The chemical reduction of FeCl_3_ using NaBH_4_ or KBH_4_ as a reducing agent is the most bottom-up method used to obtain nZVI, which subsequently is used for phosphate removal (Table 1). Maamoun et al. [30] obtained nZVI with a particle size of about 44 nm and a BET-specific surface area of 43.0 m^2^ g^−1^ using KBH_4_. According to adsorption isotherms at pH = 7, nZVI showed a maximum adsorption capacity (q_max_) of 54.3 mg g^−1^. Meanwhile, Shanableh et al. [31] obtained nZVI with a particle size between 74 and 186 nm using the same reducing agent, which at pH = 7 showed a q_max_ of 186.5 mg g^−1^. The pristine nZVI had special structures and abundant reactive surface sites for phosphate removal due to its small particle size and large specific surface area. However, nZVI, due to magnetic and van der Waals forces, tends to aggregate quickly, forming chain-like aggregate structures [51], resulting in a negative effect on the effective surface area, reaction sites, and its efficiency at removing phosphate [52,53]. In addition, NaBH_4_ is an expensive and dangerous chemical which produces H_2_ (flammable), and during the synthesis of nZVI, oxidized boron species produced cannot be easily separated from the final NPs.

In several countries, those oxidized boron species are strictly prohibited from entering bodies of water [27]. As a solution to this problem, green biological synthesis methods have been proposed to reduce Fe^3+^ to Fe^0^. This kind of method is based on plant extracts, which are non-toxic, inexpensive, and simple [48]. Various plants have been used to obtain pristine nZVI and nanocomposites [54], including green tea [55], sorghum bran [56], eucalyptus leaves [57], banana, mango, and pomegranate [49]. The plant extracts have a high concentration of polyphenols, which can act as reducing or/and coating/stabilizing agents, enhancing the electrostatic repulsion and steric hindrance of NPs, and decreasing their aggregation. The NPs obtained by green synthesis methods have a more defined shape and sometimes a smaller particle size than other chemical synthesis methods (Table 1) [58,59]. Soliemanzadeh et al. [59] synthesized nZVI using pistachio green hulls (P-nZVI) and leaves of Shirazi thyme (Th-nZVI) as both capping and reducing agents. The green nZVI showed a particle size of 40–70 nm. From the Langmuir model, q_max_ values of 29.33 and 40.52 mg g^−1^ were determined for P-nZVI and Th-nZVI, respectively. The mechanism proposed for phosphate removal by NPs was physical and chemical adsorption and precipitation due to Fe^2+^ cations released from nZVI. The main advantage of using plant residues as reducing agents is a decrease in the production costs of NPs.

## 3. Nanocomposites

### 3.1. nZVI Stabilized with Organic Molecules

The organic polymers and long-chain organic molecules are environmentally friendly and provide steric and electrostatic repulsion, preventing aggregation of nZVI. Carboxymethyl cellulose sodium (CMC), polyacrylamide (PAA), polyethylene sorbitan monolaurate (PSM), polyvinylpyrrolidone (PVP), ostrich bone waste (OBW), cellulose filter paper, sugarcane bagasse (SCB) chitosan (CS), and starch, among others, have been used to stabilize nZVI, which were later used to remove phosphate (Table 2).

Eljamal et al. [36] synthesized nZVI with CMC, PAA, PSM, and PVP. Subsequently, they determined that the CMC-nZVI nanocomposite showed a smaller size (9.53 nm) than individual nZVI particles (65.4 nm), but the q_max_ values shown by nZVI and CMC-nZVI were 61 and 56.8 mg g^−1^, respectively. The decrease in phosphate adsorption by CMC-nZVI was associated with electrostatic repulsion between nanocomposite and phosphate anions. Therefore, the smaller particle size of NPs is not the key factor contributing to the improvement in adsorption capacity.

### 3.2. Encapsulated nZVI 

The encapsulation of nZVI has emerged to improve its stability, suspension, and transport in terrestrial systems. Calcium alginate and few-layered graphene have been used to encapsulate nZVI and then applied for phosphate removal (Table 2). Alginate is a polymer that contains folds (pore size 3.17 to 5.07 nm), allowing pollutants such as phosphate to diffuse into the beads and come into contact with pristine nZVI [71]. Mahmoud et al. [24] determined that phosphate removal was about 42% using an initial concentration of 10 mg L^−1^, pH = 6.5, a dosage of encapsulated nanoscale nZVI of 20 g L^−1^, and a stirring rate of 100 rpm for 30 min. Recently, Zhang et al. [25] synthesized nZVI encapsulated with few-layered graphene (FLG@Fe^0^). The shell formed as 1–3 layers of turbostratic stacked graphene (G) nanosheets with an interlayer spacing between 0.36 and 0.38 nm. Due to the G shell protecting nZVI cores from fast passivation, FLG@Fe^0^ exhibited a phosphate adsorption capacity of 214.7 mg g^−1^.

### 3.3. nZVI Supported/Immobilized on Organic or Inorganic Materials 

Organic and inorganic materials such as biochar (BC), clays, and minerals have been widely used as a technology in the remediation of polluted waters and as support for the adsorbent. This is because they have a wide and diverse range of unique characteristics, including (i) low cost, (ii) large specific surface area, (iii) highly porous structure, (iv) ready availability, and (v) long-lasting performance. Although several studies have supported nZVI in BC to remove anionic pollutants, only studies carried out by Ma et al. [63] and Ai et al. [65] have immobilized nZVI in rape straw BC (RSBC) and sulfur-BC (S@BC), respectively, and subsequently used it to remove phosphate. According to the Sips isotherm model at pH = 7, nZVI/RSBC nanocomposite showed a q_max_ 4.48 times higher than RSBC [63]. The high phosphate removal by nanocomposite was associated with electrostatic attraction and hydrogen bonding between absorbate and positively charged adsorption sites of the adsorbent.

On the other hand, nZVI used to remove phosphate anions has been supported in inorganic clays such as bentonite (B) and zeolite (Z) (Table 2) [59], and inorganic minerals such as apatite [68]. For example, Soliemanzadeh and Fekri [59] determined that phosphate adsorption by B-nZVI was 5.99 times higher than by B. They suggested that outer-sphere surface complexation was the dominant mechanism of reaction between phosphate and B, while inner-sphere binding and covalent bonds were involved in the interaction between phosphate and the B-nZVI surface [59]. These studies suggest that a synergistic effect is generated when nZVI is mixed with inorganic and organic materials.

### 3.4. Bimetallic nZVI

In the bimetallic nZVI systems, Fe^0^ acts as an electron donor, while noble metals such as Cu, Ni, and Pd act as catalysts to promote the transformation of H_2_ into atomic H, thereby improving the reduction reactivity of nZVI [70]. In comparison with pristine nZVI, bimetallic NPs show faster reaction kinetics and reduced formation of toxic intermediates deposited on the surface of NPs [72].

Only nZVI mixed with metals such as Cu and Ni have been used in phosphate removal (Table 2). Takami et al. [73] found that phosphate removal by nZVI and bimetallic Fe/Cu NPs was 91.37 and 94.96%, respectively. The slight increase in phosphate adsorption by bimetallic NPs was associated with a greater number of active sites and a decreased solution pH. A decrease in pH values caused an increase in positive surface charge, allowing the nanocomposite surface to be more suitable for the adsorption of phosphate anions. After synthesizing Fe–Ag bimetallic NPs, Marková et al. [74] found that, although Ag did not contribute to improving phosphate adsorption, a double shell formed by magnetite/lepidocrocite (Fe_3_O_4_/γFeOOH) on the surface of the nZVI was responsible for the low oxidation of NPs by oxygen (O_2_). Moreover, due to bimetallic NPs based on Ag, an element with a high antimicrobial capacity, the authors proposed these bimetallic NPs to purify water contaminated with various bacteria.

In addition, recent investigations have immobilized bimetallic Fe^0^ NPs in organic molecules and mineral clays [69]. Shen et al. [69] observed that chelating resin DOW3N Fe/Ni (D-Fe/Ni) and chelating resin DOW3N-Fe/Cu (D-Fe/Cu) could interact with phosphate in the presence of nitrate through mechanisms of reduction, adsorption, and co-precipitation without affecting nitrate reduction. In addition, based on X-ray photoelectron spectroscopy (XPS) analysis, phosphate adsorption included a polymeric ligand exchange. Moreover, the chelating resin DOW3N improved the dispersion of bimetallic NPs and their reactivity by serving as pre-concentrated reagents and mediating the electron transfer reaction.

## 4. Experimental Conditions on Phosphate Adsorption Efficiency 

### 4.1. Effect of Initial Solution pH 

The solution pH influences the charge of nZVI particles and phosphate dissociation. It has been shown that at an acidic pH, phosphate is adsorbed by nZVI through an electrostatic attraction FeOOH ≡ PO_4_^3−^ → FeOOH ≡ PO_4_ and complex formation [39]. At a ≤ 7, nZVI is easily oxidized and forms a shell core of Fe oxides/hydroxides such as lepidocrocite, maghemite, goethite, ferrihydrite, and magnetite. Sleiman et al. [75] showed that phosphate, due to its negative charge, was adsorbed on positively charged iron oxides such as maghemite/magnetite and lepidocrocite, the isoelectric point (IEP) of which varies between 6.5 and 9.0. All those iron oxides showed a high affinity for phosphate. The release of Fe^2+^/Fe^3+^ ions from nZVI contributes to high phosphate removal through simultaneous coagulation/precipitation and co-precipitation processes [29,76]. Liu et al. [28], using 5 mg L^−1^ of initial phosphate concentration in slightly acidic aqueous media, determined a removal higher than 99%. This result was mainly associated with the corrosion process of nZVI resulting in iron oxide formation and subsequent removal of phosphate. They proposed four reactions for phosphate removal.

(1) $${\;\mathrm{Fe}}^{2+}+{\mathrm{PO}}_{4}^{3-}\to {\mathrm{Fe}}_{3}{\left({\mathrm{PO}}_{4}\right)}_{2\left(\mathrm{pp}\right)}$$

(2) $${\mathrm{Fe}}^{3+}+{\mathrm{PO}}_{4}^{3-}\to {\mathrm{FePO}}_{4\left(\mathrm{pp}\right)}\;$$

(3) $$\mathrm{Fe}{\left(\mathrm{OH}\right)}_{2}+{\mathrm{PO}}_{4}^{3-}\to \mathrm{Fe}{\left(\mathrm{OH}\right)}_{2}\equiv {\mathrm{PO}}_{4}$$

(4) $$\mathrm{Fe}{\left(\mathrm{OH}\right)}_{3}+{\mathrm{PO}}_{4}^{3-}\to \mathrm{Fe}{\left(\mathrm{OH}\right)}_{4}\equiv {\mathrm{PO}}_{4}$$

Studies have shown that the formation of precipitate Fe_3_(PO_4_)_2_·H_2_O_(pp)_ (K_sp_ = 9.94 × 10^−29^) and FePO_4(pp)_ (K_sp_ = 1.3 × 10^−22^), and mineral phases of vivianite (Fe_3_(PO_4_)_2_·8H_2_O) are highly relevant in phosphate removal [20,28]. Due to the greater corrosion of Fe, the mineral Fe_3_(PO_4_)_2_·8H_2_O is more predominant at an acidic than basic pH [33]. Li et al. [77], studying the effect of nZVI on phosphate availability in sediments, found that at an acid pH, the formation of FePO_4(pp)_ was the predominant mechanism of pollutant immobilization. By contrast, at a basic pH, it was reported that phosphate adsorption efficiency decreased for different reasons. For example, when the pH solution is higher than the IEP of nZVI, which fluctuates between 6 and 9, electrostatic repulsion occurs between the negatively charged nZVI surface and phosphate anions [44]. In the same way, electrostatic repulsion increases by dissociation of H_2_PO_4_^−^ → HPO_4_^2−^, and when there is a high amount of OH^−^ in the solution, they can compete with phosphate for adsorption sites of NPs, so the formation of precipitate between P-Fe^3+^ is inhibited due to decreased Fe^3+^ ions released from NPs [20]. A decrease in phosphate adsorption with increasing pH has also been reported for nanocomposites [31].

### 4.2. Effect of Oxygen 

Similar to acidic pH solutions, the presence of O_2_ in systems favors the corrosion process of nZVI, forming (hydro)ferric oxides and oxidation from Fe^0^ to Fe^2+^, which is later oxidized to Fe^3+^ [41]. This is represented by Equations (5)–(7).

(5) $$2{\mathrm{Fe}}^{2+}+O_{2}\to 2\mathrm{FeO}$$

(6) $$4{\mathrm{Fe}}^{0}+3O_{2}\to 2{\mathrm{Fe}}_{2}O_{3}$$

(7) $$4{\mathrm{Fe}}^{0}+3O_{2}+6H_{2}O\to 4{\mathrm{Fe}}^{3+}+12{\mathrm{OH}}^{-}$$

Iron oxy/hydroxides forming on the surface of NPs are suitable adsorbents of anionic pollutants, including AsO_4_^3−^, SeO_4_^2−^, Cr_2_O_7_^2−^, SO_4_^2−^, NO_3_^−^, and PO_4_^3−^, among others [45]. Along these lines, a study carried out by Zhang et al. [29] determined that the amount of phosphate adsorption by nZVI reached 11.1, 16, and 22.7 mg g^−1^ under the anaerobic, anoxic, and oxic systems, respectively.

### 4.3. Effect of Reaction Time 

Due to the transformations of nZVI in aerobic environments, pollutant removal efficiency is highly dependent on contact time. When the BET-specific surface area of nZVI was 27.7 m^2^ g^−1^ and the initial phosphate concentration was 10 mg L^−1^, removal proved to be rapid and easy, reaching an efficiency higher than 99% in 5 min, which remained constant up to 180 min [22]. This was associated with the high surface area of NPs, which allowed the adsorbent to have more active binding sites for phosphate adsorption [22]. Sleiman et al. [78] determined adsorption of about 35 mg g^−1^ after 8 h of aging of nZVI; however, at 6 days, adsorption decreased to 2 mg g^−1^. This significant difference was attributed to the fact that during the first few hours, the recently formed iron oxide coating favored phosphate elimination. Meanwhile, the longer aging period caused a reduction in redox activity and consequently decreased the ability to remove phosphate.

### 4.4. Effect of Ionic Strength 

The variation in the amount of phosphate adsorbed with ionic strength can be used as an indicator of the type of adsorption (physical or chemical). It has been suggested that when there is a high variation in adsorption to changes in ionic strength, phosphate interacts with nZVI mainly through physical interactions (electrostatic and van der Waals forces). However, when ionic variation has no greater relevance on adsorption, predominant interactions are chemical, which implies a covalent bonding along with ionic bonding at the solid–liquid interface [78]. Salts such as NaCl, KCl, and CaCl_2_ are commonly used to evaluate the effect of ionic strength. A null variation of phosphate adsorption by nZVI with the variation of ionic strength [22,34,78] and nanocomposites has been reported [44]. Almeelbi and Bezbaruah [34], after increasing ionic strength from 0 to 10 mM of CaCl_2_, determined that the adsorption fluctuated between 96.0 and 98.5%, which explains the high stability of the nZVI–phosphate interaction.

### 4.5. Effect of Dosage of Adsorbent

The dosage of nZVI-based nanomaterials has a direct effect on phosphate removal efficiency. In general, pollutant removal by nZVI is a process dependent on surface area, so it is reasonable that as the dosage of NPs increases, so too does the phosphate removal. A greater number of particles would provide a high amount of active sites on the surface of nZVI, and Fe^2+^/Fe^3+^ ions released into the solution, which would favor adsorption and co-precipitation processes with phosphate [33,79]. This situation is in line with research carried out by Almeelbi and Bezbaruah [34], who, after increasing the initial dose of nZVI from 80 to 560 mg L^−1^, determined that phosphate removal increased by 78%. These results were attributed to an increase in electrostatic interactions. On the other hand, some studies have found that anion removal efficiency decreased at high nZVI doses [47]. This is due to an increase in the aggregation of nZVI, resulting in a decrease in the BET-specific surface area and consequently affecting the number of adsorption sites. Thus, an optimal dose of nZVI-based materials for phosphate remediation must be found.

### 4.6. Effect of Initial Concentration of Phosphate 

For a fixed amount of adsorbent at a high initial concentration of phosphate, the removal percentage decreases due to a high ratio of phosphate concentration at active adsorption sites on the nZVI surface, leading to the saturation of adsorbent surface and the decrease in adsorbent–adsorbate interactions [22,33]. Maamoun et al. [39], using 1 g L^−1^ of nZVI and phosphate concentrations of 50 and 100 mg L^−1^, determined that the nZVI had a phosphate removal efficiency of 94.2 and 76.8%, respectively. Similarly, Mahmoud et al. [24], using nZVI/Ca encapsulated, a dose of nZVI: 20 g L^−1^, an initial phosphate concentration of 1 mg L^−1^ at pH = 6.5, and a stirring rate of 100 rpm for a contact time of 30 min, found that removal was 66% and decreased to 42% when its concentration was increased to 10 mg L^−1^. Additionally, initial phosphate concentration increases may also involve a greater aggregation of nZVI, causing a reduction in the number of active sites of NPs, and decreasing phosphate adsorption. However, this effect can be dependent on the nZVI nanocomposite [28,39,64]. Arshadi et al. [64], using an initial concentration of 100 mg L^−1^ for phosphate, determined a poor adsorbate removal by nZVI. In the meantime, after immobilization of nZVI on OBW, the NPs showed a good dispersion, reaching a removal of 97.7%, 91%, and 32.6%, when initial phosphate concentration was 30, 110, and 1000 mg L^−1^, respectively [64].

### 4.7. Interferences 

In wastewater and groundwater sources, anions such as chloride (Cl^−^), bicarbonate (HCO_3_^−^), thiosulphate (S_2_O_3_^2−^), NO_3_^−^, SO_4_^2−^, and dissolved organic matter (DOM) can compete with phosphate for adsorption sites of the adsorbent, affecting nZVI-based nanomaterials adsorption capacity [64]. The information reported on the behavior of nZVI is diverse. Using various concentrations of NO_3_^−^ (1, 5, and 10 mg L^−1^) and SO_4_^2−^ (100 and 500 mg L^−1^), Almeelbi and Bezbaruah [34] determined that phosphate removal by nZVI from an aqueous solution decreased with increasing concentrations of the interferents. This contrasts with results obtained by [2,26,29,35,61]. Zhang et al. [29], after evaluating the effect of anions such as SO_4_^2−^, S_2_O_3_^2−^, Cl^−^, and NO_3_^−^ in a concentration range from 0 to 0.2 mmol L^−1^, determined an increase in phosphate adsorption by nZVI. The reason was related to the accelerated corrosion of Fe^0^ caused by anions, promoting the formation of a secondary phase. Similarly, Wan et al. [2] used 0.5 g L^−1^ of Fe^0^/Fe_3_O_4_ nanocomposite, 0.4 mg L^−1^ of phosphate, and concentrations of Cl^−^, SO_4_^2−^, NO_3_^−^, and HCO_3_^−^ common in river water to demonstrate that anions increase phosphate adsorption by Fe^0^/Fe_3_O_4_. These results were associated with improved electron transfer from Fe^0^ to the surface of Fe_3_O_4_, accelerating the corrosion rate of Fe^0^. Shen et al. [69], after using nanocomposites of D-Fe/Cu and D-Fe/Ni and an initial phosphate, and NO_3_^−^ concentrations of 5 and 20 mg L^−1^, respectively, determined that NO_3_^−^ removal by the NPs D-Fe/Cu and D-Fe/Ni was 95.5% and 98.7%, respectively, and phosphate removal was 93.0% and 99.0%, respectively. From this study, Shen et al. [69] concluded that phosphate adsorption was promoted by the presence of NO_3_^−^, because metal (Cu, Ni, or Fe) was oxidized/corroded during the reduction of NO_3_^−^, which resulted in a generation of more metal oxides/hydroxides on the surface of NPs, which can react with phosphate through adsorption and co-precipitation processes.

The effect of DOM as an interferer has been evaluated using organic molecules such as cationic chitosan (CS), humic acid (HA), and fulvic acid (FH). According to the Langmuir model, Bhattacharjee et al. [32] found for nZVI a decrease in the q_max_ from 523 to 342 mg g^−1^ at pH = 4.5 in the presence of CS, while at pH = 6.5, CS had the opposite effect, reaching a removal close to 770 mg g^−1^. Meanwhile, in the presence of HA at pH = 4.5 and 6.5, the q_max_ increased to 698 and 797 mg g^−1^, respectively. The differences between the two interferents were associated with molecular weight and surface charge.

### 4.8. Regeneration

The repeated use of an adsorbing material is highly relevant due to reducing the costs involved, making the spent adsorbent economically viable [80]. Regeneration methods include chemical extraction, heat treatment, supercritical regeneration, bio-regeneration, ultrasonic regeneration and electro-Fenton regeneration. Arshadi et al. [64] evaluated the reuse of an ostrich bone waste-nZVI (OBW-HNO_3_-nZVI) nanocomposite using 1.0 mol L^−1^ of NaOH as the chemical agent to regenerate active surface sites, and showed that the nanocomposite maintains its phosphate removal ability even after twelve adsorption–desorption cycles. Similarly, Arshadi et al. [60] studied the reuse of stabilized nZVI on cellulose filter paper (FP-OH-nZVI) using 1.0 mol L^−1^ NaOH to regenerate adsorption sites. They found that the removal capacity was greater than 99%, remaining practically constant after seven adsorption–desorption cycles. This study demonstrated that phosphate adsorption capacity by FP-OH-nZVI was practically not altered after being regenerated with NaOH.

### 4.9. Temperature 

Several studies have shown that an increase in temperature (°T) improves the accessibility of phosphate at the active sites of nZVI and nanocomposites due to the expansion of pore size and the activation of the nZVI-based nanomaterials surface, which implies an increase in the amount of phosphate removed in single-component and competition systems [47,60,81]. Anastopoulos et al. [81] used °T studies to calculate thermodynamic parameters such as enthalpy (ΔH^0^), entropy (ΔS^0^), and Gibbs free energy (ΔG^0^). Zheng et al. [38] showed that when °T increased from 35 to 50 °C, the q_max_ for phosphate by nZVI increased from 51.99 to 54.40 mg g^−1^, respectively. In addition, ∆H^0^ and ∆S^0^ were positive, indicating that the phosphate adsorption process was spontaneous and favorable, respectively. Maamoun et al. [39] reported an increase of about 33% in phosphate adsorption by nZVI when °T increased from 15 to 70 °C, and a spontaneous adsorption process as evident from the ΔH^0^ value of 67.87 kJ mol^−1^. Moreover, they indicate that adsorption by nZVI may involve both chemisorption and physisorption processes. This behavior has also been demonstrated for OBW-HNO_3_-nZVI [64] and FP-OH-nZVI nanocomposites [60]. However, these results are dependent on nZVI and the type of nanocomposite. Almeelbi and Bezbaruah [34] evaluated the effect of 4, 22, and 60 °C on the removal of phosphate by nZVI, which was between 91.4 and 95.3%, although there were no significant differences between these values. The authors indicated that it was associated with rapid phosphate removal, which takes place within the first 10 min and may be the reason why no distinction could be made between removal achieved at three different °T [34]. For their part, Chen et al. [44], using starch-stabilized nZVI, found phosphate adsorption decreased from 98.72 to 88.26 mg g^−1^ with an increase from 10 to 40 °C, respectively. However, they did not discuss the reasons for the decreased phosphate adsorption with °T.

## 5. Relationship between Maximum Adsorption Capacity and Partition Coefficient

In laboratories, phosphate adsorption isotherms in batch systems and subsequent experimental data fitted to the Sips, Redlich–Peterson, Langmuir, Temkin, and Freundlich mathematical models have made it possible to determine parameters such as intensity adsorption (n), affinity (K_L_), maximum binding energy (A_T_), and maximum adsorption capacity (q_max_). In general, studies have proposed an adsorbent to remove pollutants based mostly on the q_max_ value [34,44,48,82]. Nevertheless, Szulejko et al. [83] indicated that q_max_ is dependent on initial concentration and adsorbent dose, which leads to erroneous conclusions. They suggested that partition coefficient (PC) is a better parameter because it can help reduce deviation in evaluating the performance of adsorbents in different experimental conditions, since it not only considers q_max_ values but is also based on practical adsorbent performance. The PC can be obtained by Equation (8) [84].

(8) $$\mathrm{PC}=\frac{\mathrm{Adsorption}\;\mathrm{capacity}}{\mathrm{Initial}\;\mathrm{concentration}*\left(1-\mathrm{removal}\;\mathrm{rate}\right)}$$

where adsorption capacity refers to phosphate adsorption capacity (mg g^−1^), initial concentration refers to initial phosphate concentration (µM), and removal rate refers to phosphate removal efficiency (%). 

The PC parameter contributes to studying the application of nanoadsorbents and determining if they are suitable for use in the environment [85]. Table 3 shows the PC values where nZVI and its nanocomposites are used to remove phosphate. However, in some articles, the information necessary to determine the PC values is not shown. According to our results, we found that the q_max_ was not related to PC values. Therefore, some researchers might have concluded incorrectly the likely most effective material to remove phosphate from aquatic systems. Based on Table 3, nZVI synthesized by Shanableh et al. [31] and starch-nZVI nanocomposite had the highest adsorption. Nevertheless, the highest PC value was shown by sepiolite-nZVI. Therefore, considering the cost-efficiency relationship and PC values, we suggest the use of nanocomposites based on sepiolite clays as the most promising nanomaterial for phosphate adsorption.

## 6. Risks and (Dis)advantages Associated with the Use of nZVI

We should use sustainable technologies to remediate aqueous or contaminated soil systems that are easy to implement and do not generate toxic effects on people’s health and the environment. The main problems with the use of nZVI and its nanocomposites on a large scale are related to their aggregation [86] as well as limited field studies, creating a lack of knowledge about their real impact on the environment. Specifically, NPs released into aquatic systems and soils will be absorbed by microorganisms and plants, allowing their incorporation into the trophic chain. This could be highly dangerous because several studies have reported on the adverse effects of NPs on human health. As a consequence, NPs have been classified as emerging pollutants [87]. Research on nZVI has shown lower toxic effects than other NPs, such as Cu and Ag [23]. Various laboratories and companies provide different types of nZVI used to remove or immobilize various pollutants from groundwater and soil systems [88]. It has been suggested that the adsorbent-based technology of nZVI is an up-and-coming area for maintaining a balance in the environment [14]. Although nZVI is not being used on a large scale to immobilize phosphate in aquatic and soil systems, it has been used for organic element compounds (pesticides, polychlorinated hydrocarbons, chlorobenzenes, and coloring agents) and potentially toxic elements (Cu, Ni, Cd, Pb, and Zn) [89,90], showing its potential benefit to preserve the environment. For example, contaminated groundwater is in geographic locations that are difficult to access with conventional treatments, and expensive and time-consuming to install. In this context, Chowdhury et al. [89] injected 142 L of CMC stabilized monometallic nZVI synthesized in groundwater to decrease trichloroethylene (TCE) pollutant concentrations. They determined more than 99% of the TCE concentrations were eliminated, and it was shown that NPs could travel long distances in groundwater.

## 7. Summary

As discussed in the above sections, to determine the best parameters and conditions to achieve phosphate adsorption by nZVI, it is necessary to consider together different aspects, for example: (i) the synthetic methods can involve high costs, adequate instruments, the generation of secondary toxic products, diverse morphology and physic-chemistry surface characteristics, and others. In this context, the utilization of green synthesis by bottom-up methods is an excellent alternative to produce non-toxic NPs with good surface characteristics. In addition, the immobilization of nZVI particles in solid substrates allows the stabilization and dispersion of them, favoring their efficiency; (ii) regarding experimental conditions on phosphate adsorption efficiency, the pH is an important parameter because it strongly affects the adsorption capacity of the material and speciation of phosphate anions. Therefore, it is important to know the isoelectric point of the substrate to avoid electrostatic repulsions between the adsorbate and the adsorbent, and thus favor adsorption; (iii) other parameters such as reaction time, ionic strength, the dosage of adsorbent, initial concentration of phosphate, and °T should be considered in the adsorption process because they have an influence on spontaneity, active site, affinity, and interaction between nZVI and the phosphate; and finally, iv) it is necessary to take into consideration that for soil and water application, the risks and (dis)advantages associated with the use of nZVI should be known so as not to generate secondary damage or contamination.

## 8. Conclusions and Projections

Phosphate removal by nanoscale zero-valent iron (nZVI) from aquatic systems has been extensively tested at the laboratory level in different experimental conditions, offering very promising results to solve problems on a global scale. However, there are various problems and uncertainties with the synthesis of nZVI materials and their practical applications in the removal of phosphate from aqueous systems. Therefore, it is necessary to explore new synthesis technologies to produce materials that can capture nZVI particles with similar characteristics regardless of where they were manufactured. In general, we found the immobilization of nZVI in solid substrates presents high stability and dispersion, though it is not always associated with higher phosphate adsorption. In addition, studies on phosphate adsorption should report details of experimental conditions as well as adsorption capacity and partition coefficient (PC) values. In this context, sepiolite-nZVI shows the highest PC value; therefore, it is considered the most effective material to remove phosphate from aquatic systems. Although nZVI-based nanomaterials have been used extensively to remove phosphate from aquatic systems, their potential environmental risks may not be fully included in current studies. For this reason, future studies should investigate the applications of pristine nZVI and its nanocomposites for phosphate removal under natural environmental conditions. In addition, experimental studies of nZVI-based nanomaterials must assess their impact on humans and environmental systems. 

## Figures and Tables

**Table 1:** Synthesis and properties of nZVI and mechanisms involved in phosphate removal.

Synthesis nZVI	Preparation	Washes	Particle Size (nm)	BET-Area (m^2^ g^−1^)	Interaction or Mechanisms	References
	Top-Down Methods					
Mechanical	Goethite particle sizes (300–850, 125–300, 96–125, 75–96, and <75 μm) + 550 °C.	-	80–150	19.9–22.8	Precipitated of FePO_4_	[28]
	Limonite < 74 μm + °T 300, 400, 450, 500, 550, and 600 °C	-	-	37.52–17.24	Precipitation and adsorption	[29]
	Bottom-Up Methods					
Chemical	FeCl_3_·6H_2_O + NaBH_4_	Deionized (DI) water (H_2_O)	-	27.65	Adsorption and co-precipitation	[22]
	FeCl_3_·H_2_O + NaBH_4_	Three times by DI H_2_O and ethanol	44	43.09	Adsorption and co-precipitation	[30]
	FeSO_4_·7H_2_O + KBH_4_	Three times by DI H_2_O and ethanol	74–186	-	Adsorption, ion exchange, and precipitation	[31]
	FeSO_4_·7H_2_O + KBH_4_	Three times by H_2_O and ethanol	30–300	15	Electrostatic adsorption, ion exchange, and surface precipitation	[32]
	FeSO_4_·7H_2_O + NaBH_4_	-	30–80	20.92	Adsorption, precipitation, and exchange	[33]
	FeCl_3_ + NaBH_4_	DI H_2_O	10–30	25	Ligand exchange	[34]
	FeCl_3_·6H_2_O + NaBH_4_	Three times by DI H_2_O	40	61	Adsorption on iron (hydr)oxides	[35]
	FeCl_3_ + NaBH_4_	Three times by DI H_2_O	65.4	-	Chemical bonds	[36]
	FeCl_3_ + NaBH_4_	Three times by DI H_2_O	40–150		Surface complexation, co-precipitation	[37]
	Bought from Aladdin Industrial Corporation Company	-	20−100	4.66	Chemical interaction	[38]
	FeCl_3_.6H_2_O + NaBH_4_	Three times by DI H_2_O and ethanol	-	-	Electrostatic attraction and co-precipitation	[39]
	FeSO_4_·7H_2_O + KBH_4_	-	100	-	Physical adsorption, precipitated of Fe_3_(PO_4_)_2_·8H_2_O	[40]
	FeSO_4_·7H_2_O + KBH_4_	DI H_2_O	-	32.38	Chemical adsorption and precipitated of Fe_3_(PO_4_)_2_	[41]
	FeCl_3_·6H_2_O + NaBH_4_	DI H_2_O	-	-	Electrostatic adsorption	[42]
	FeCl_3_·6H_2_O + NaBH_4_	Ethanol	35	-	Electrostatic attraction and repulsion	[43]
	FeCl_3_·6H_2_O + KBH_4_	DI H_2_O and anhydrous ethanol	78	16.57	Adsorption by inner-sphere surface complexes	[44]
	FeCl_3_·6H_2_O + NaBH_4_	Three times by DI H_2_O	44	17.32	Adsorption on iron (hydr)oxides, co-precipitation	[45]
	FeCl_3_·6H_2_O + NaBH_4_	Three times by DI H_2_O and ethanol	13	32.38	Physical adsorption and chemical adsorption	[46]
	FeCl_3_·6H_2_O + NaBH_4_	Ethanol	50	143.163	Electrostatic attraction and co-precipitation	[47]
	FeSO_4_·7H_2_O + leaves of Shirazi thyme FeSO_4_·7H_2_O + pistachio green hulls	Ethylene	40–70	-	Physical and chemical adsorption on iron (hydr)oxides and precipitation	[48]
Green biological	FeCl_3_ + pomegranate (GP)	-	114	-	Electrostatic adsorption and surface complexation	[49]
	FeCl_3_ + banana (BP)		96			
	FeCl_3_ + mango (MP)		75			
	FeCl_3_·6H_2_O + black tea	Ethanol	-	-	Binding	[50]

**Table 2:** Properties of nanocomposites of nZVI and mechanisms for phosphate removal.

Adsorbent	Preparation Materials	Washes	Particle Size (nm)	BET-Area (m^2^ g^−1^)	Interaction Mechanisms or Reactions with Phosphate	References
	nZVI Stabilized with Organic Molecules					
Chitosan-nZVI	FeSO_4_·7H_2_O + KBH_4_ + medium molecular weight chitosan	-	117–200	-	Adsorption, ion exchange, and precipitation	[31]
CMC-nZVI	FeSO_4_·7H_2_O + NaBH_4_ + CMC	-	-	-	-	[33]
CMC-nZVI	FeCl_3_ + NaBH_4_ + polymer (CMC, PAA, PSM, and PVP)	Three times by DI H_2_O	9.53	-	Chemical bonds	[36]
PAA-nZVI			106.4			
PSM-nZVI			106.6			
PVP-ZVI			108.8			
Cation exchange resin-nZVI	FeSO_4_·7H_2_O + KBH_4_ + strong acid cation exchange resin	DI H_2_O	-	0.13	Chemical adsorption and precipitated of Fe_3_(PO_4_)_2_	[41]
Starch-nZVI	FeCl_3_·6H_2_O + KBH_4_ + starch	DI H_2_O and anhydrous ethanol	43	35.28	Adsorption by inner-sphere surface complexes	[44]
Cellulose filter paper-nZVI	Cellulose modified with NaOH + FeCl_3_ anhydrous + NaBH_4_	H_2_O and ethanol	<30	-	Chemisorption, electrostatic attraction, and precipitated of Fe_3_(PO_4_)_2_·8H_2_O	[60]
SCB-nZVI)	Sugarcane bagasse + FeCl_3_ + KBH_4_	Four times by ethyl alcohol	150–300	-	Electrostatic sorption and formation of inner spherical complex	[61]
	nZVI Encapsulated					
Alginate beads-nZVI	FeCl_3_·6H_2_O + NaBH_4_ + sodium alginate	-	30–55	-	Adsorption and chemical precipitation	[24]
Few-layered graphene-nZVI	Fe(NO_3_)_3_⋅9H_2_O + lignin + tetrahydrofuran/water	-	5–15	10	Co-precipitation and mono- and/or bidentate chemisorption interactions	[25]
Alginate biopolymer (Ag)-nZVI	Sodium alginate + nZVI + CaCl_2_ solution	-	-	-	Electrostatic interaction	[42]
Alginate biopolymer-nZVI	Sodium alginate + nZVI + CaCl_2_ solution	-	-	-	-	[43]
	nZVI Supported on Organic Materials					
Graphene oxides-nZVI	FeSO_4_·7H_2_O + graphitic oxide + NaBH_4_	-	15	-	Physical adsorption, precipitated of Fe_3_(PO_4_)_2_·8H_2_O	[40]
Activated carbon-nZVI	FeCl_3_ + activated carbon +NaBH_4_	Three times by ethanol	20–60	88.29	-	[62]
Rape straw biochar-nZVI	FeSO_4_ + rape straw biochar (RSBC) + NaBH_4_	Three times by ethanol	20–30	34.23	Complexation, hydrogen bonding, and electrostatic interaction	[63]
OBW-HNO_3_-nZVI)	FeCl_2_·4H_2_O + ostrich bone waste-HNO_3_ + NaBH_4_	-	5–30	41.4	Chemisorption and precipitated of Fe_3_(PO_4_)_2_·8H_2_O	[64]
Sulfur-nZVI@biochar	Biochar + sulfur powder + iron powder	-	-	-	Electrostatic attraction, surface chemical precipitation, hydrogen bonding, and ligand effects	[65]
Activated carbon/nZVI	FeCl_3_·6H_2_O +activated carbon + NaBH_4_	DI H_2_O	<50	72.66	Adsorption and co-precipitation	[66]
	nZVI Supported on Inorganic Minerals					
Zeolite 1-Nano	nZVI + sodium zeolite	-	-	54.33	Chemical adsorption and precipitated of KFeH_14_(PO_4_)_8_·4H_2_O	[46]
Zeolite 2-Nano	FeSO_4_ + sodium zeolite + nZVI	-	-	29.01		
Bentonite-nZVI	Bentonite + iron + green tea	Ethanol	8–30	32.54	Chemisorption	[54]
Bentonite-nZVI	FeSO_4_·7H_2_O + leaves of green tea + natural bentonite	Ethylene	40–60	-	Inner-sphere binding and covalent bonds	[59]
Sepiolite-nZVI	FeCl_3_ + acid modified sepiolite + NaBH_4_	Three times by ethanol	20–70	-	Electrostatic interaction and co-precipitation	[67]
Bio-apatite-nZVI	Bio-apatite + FeCl_3_·6H_2_O + NaBH_4_	Ethanol	20 to 60	109	Fe_3_(PO_4_)_2_·8H_2_O	[68]
	nZVI Bimetallic					
FeCl_3_·6H_2_O + CuCl_2_ + NaBH_4_	FeCl_3_·6H_2_O + CuCl_2_ + NaBH_4_	Three times by DI H_2_O	24	32.4	Adsorption on iron (hydr)oxides, co-precipitation	[45]
Chelating resin DOW3N-Fe/Cu (D-Fe/Cu)	Fe_2_(SO_4_)_3_ + CuCl_2_·2H_2_O + NaBH_4_ + chelating resin DOW3N	DI H_2_O and anhydrous ethanol	-	-	Reduction, adsorption, co-precipitation, and a newly formed adsorbent-polymeric ligand exchanger	[69]
Chelating resin DOW3N Fe/Ni (D-Fe/Ni)	Fe_2_(SO_4_)_3_ + NiCl_2_·6H_2_O + NaBH_4_ + chelating resin DOW3N		-	-		
Zeolite-Fe/Ni (Z-Fe/Ni)	FeCl_3_·6H_2_O + NiCl_2_·6H_2_O + acid-activated zeolite + NaBH_4_	Three times by H_2_O and ethanol	20–30	59.2	Reduction and complexation	[70]

**Table 3:** Performance of nZVI and its nanocomposites for adsorption of phosphate from aqueous solutions.

Adsorbent	Best-Fitted Isotherm Model	°T	pH	IC (mg L^−1^)	IC (µM)	Removal Efficiency (%)	q_max_ (mg g^−1^)	PC (mg g^−1^ µM^−1^)	References
	Freundlich	25	-	1000	32,289.3	94	245.6	0.127	[22]
	Langmuir	25	7	1000	32,289.3	5.4	54.3	0.002	[30]
nZVI	Langmuir	25	5	175	5650.6	60.7	437.4	0.197	[31]
	Langmuir		7	175	5650.6	24.4	168.5	0.039	
	Langmuir	-	4.5	500	16,144.7	29.9	523	0.046	[32]
	Langmuir		6.5	500	16,144.7	23.5	412	0.033	
	-		6	90	2906.0	51.4	115.6	0.082	[33]
	-	25	7	1000	32,289.3	7.6	61.1	0.002	[36]
	Langmuir	20	7	6	193.7	88	32.4	1.393	[38]
	Langmuir	25	7	100	3228.9	76.8	76.8	0.103	[39]
	Freundlich	20	-	20	645.8	39.1	15.6	0.04	[40]
	Langmuir	10	4	440	14,207.3	56.2	247.3	0.04	[44]
	Nanocomposite								
	Organic								
Chitosan-nZVI	Langmuir	25	5	175	5650.6	49.6	289.2	0.101	[31]
			7			26.1	152.3	0.036	
CMC-nZVI	Langmuir	25	7	1000	32,289.3	7.1	56.8	0.002	[36]
PAA-nZVI						9.75	78	0.003	
PSM-nZVI						7.5	60	0.002	
PVP-nZVI						7.3	58.4	0.002	
Pistachio green hulls-nZVI	Redlich–Peterson	room	5	300	9686.8	23.61	29.3	0.004	[48]
Shirazi thyme leaf-nZVI					9686.8	35.79	40.5	0.007	
SCB/nZVI	Langmuir		5.3	200	6457.9	41.04	205.2	0.216	[61]
	Encapsulated								
Alginate beads-nZVI	-	-	-	10	322.9	62.4	0.312	0.003	[24]
	Inorganic								
Graphene oxides-nZVI	Freundlich	20	-	20	645.8	36.8	14.7	0.036	[40]
Starch-nZVI	Langmuir	10	4	440	14,207.3	73.2	322.4	0.085	[44]
Bentonite-nZVI	Sips	room	-	900	29,060.4	32.6	58.6	0.003	[54]
Bentonite-nZVI	Redlich–Peterson	-	-	500	16,144.7	27.6	27.6	0.002	[59]
Biochar-nZVI	Sips	25	7	60	1937.4	40.5	12.1	0.011	[63]
Ostrich bone waste-HNO_3_-nZVI	Langmuir	25	5	1000	32,289.3	32.6	326	0.015	[64]
Sepiolite-nZVI	Freundlich	-	4.5	25	807.2	99.4	16	3.477	[67]

## Data Availability

Not applicable.
